# Observed 3D Structure, Generation, and Dissipation of Oceanic Mesoscale Eddies in the South China Sea

**DOI:** 10.1038/srep24349

**Published:** 2016-04-14

**Authors:** Zhiwei Zhang, Jiwei Tian, Bo Qiu, Wei Zhao, Ping Chang, Dexing Wu, Xiuquan Wan

**Affiliations:** 1 Physical Oceanography Laboratory/Qingdao Collaborative Innovation Center of Marine Science and Technology, Ocean University of China, 238 Songling Road, Qingdao 266100, P.R. China; 2 Department of Oceanography, University of Hawaii at Manoa, 1000 Pope Road, Honolulu, Hawaii 96822, USA; 3 Department of Oceanography, Texas A&M University, College Station, Texas 77843, USA

## Abstract

Oceanic mesoscale eddies with horizontal scales of 50–300 km are the most energetic form of flows in the ocean. They are the oceanic analogues of atmospheric storms and are effective transporters of heat, nutrients, dissolved carbon, and other biochemical materials in the ocean. Although oceanic eddies have been ubiquitously observed in the world oceans since 1960s, our understanding of their three-dimensional (3D) structure, generation, and dissipation remains fragmentary due to lack of systematic full water-depth measurements. To bridge this knowledge gap, we designed and conducted a multi-months field campaign, called the South China Sea Mesoscale Eddy Experiment (S-MEE), in the northern South China Sea in 2013/2014. The S-MEE for the first time captured full-depth 3D structures of an anticyclonic and cyclonic eddy pair, which are characterized by a distinct vertical tilt of their axes. By observing the eddy evolution at an upstream versus downstream location and conducting an eddy energy budget analysis, the authors further proposed that generation of submesoscale motions most likely constitutes the dominant dissipation mechanism for the observed eddies.

Oceanic mesoscale eddies, which are key transporters of oceanic materials^1^,^2^,^3^,^4^, are ubiquitously observed in the world oceans^5^. Like storms in the atmosphere seen in weather maps, they evolve both in time and space, and are highly non-stationary. As such, it has been a challenge to fully capture evolving oceanic eddies in terms of their detailed 3D structures from field observations. To overcome this challenge, composite analyses of satellite altimeter and *in-situ* data have been recently used to reconstruct the mean structures of eddies in various regions of the world oceans^6^,^7^,^8^,^9^,^10^,^11^. These studies have, however, primarily focused on the upper 1000 m ocean and our knowledge of the full-depth 3D structures of oceanic eddies remains lacking. Besides, many of these composite results are based on the assumption that all cyclonic and anticyclonic eddies share similar structures, regardless of their sizes and life stages. With this assumption, they fail to provide insightful information about the evolution of the oceanic eddies.

Along with their structures, the lifecycle, including generation and dissipation processes, of oceanic eddies is of equal dynamical interest. Generation of oceanic eddies are generally better understood. The prevailing dynamical paradigm is that the oceanic eddies are generated through hydrodynamic instabilities of ocean currents, with release of available potential and kinetic energy built up by large-scale wind and surface buoyancy fluxes^12^,^13^. In contrast, our understanding of oceanic eddy dissipation mechanisms is relatively poor. Candidate mechanisms that have been proposed include damping by ocean bottom drag^14^,^15^ and surface wind stresses^16^,^17^, radiating near-inertial waves through loss of balance^18^,^19^,^20^, generating lee-waves over small-scale bottom topography^21^,^22^,^23^,^24^, and scattering into high-wavenumber vertical modes along the ocean western boundaries^25^. Recent high-resolution numerical simulations suggest enhanced submesoscale motions (with scales of O(1–50 km)) in the presence of oceanic mesoscale eddies^26^,^27^. Transferring energy from mesoscale to submesoscale motions is, therefore, suggested as another route for the dissipation of oceanic eddies^28^,^29^. Although all the above candidates may play a role, the dominant mechanism by which oceanic eddies are dissipated remains unclear due to lack of comprehensive *in-situ* observations.

The South China Sea (SCS), one of the largest marginal seas in the Pacific Ocean (Fig. 1a), is abundant with strong eddy activities as revealed by both observational and modelling studies^30^,^31^,^32^,^33^. It serves as an ideal testbed for investigating spatial structure and lifecycle of oceanic eddies. Using the *in-situ* data collected from the S-MEE, this study reports the observed 3D structures and generation processes of an anticyclonic and cyclonic eddy pair in the northern SCS (Fig. 1b–g) and tempts to quantify the eddy dissipation processes through the synergetic use of concurrent moored, ship-based, and satellite-derived observations.

## Results

### Observations

**Figure 1:**
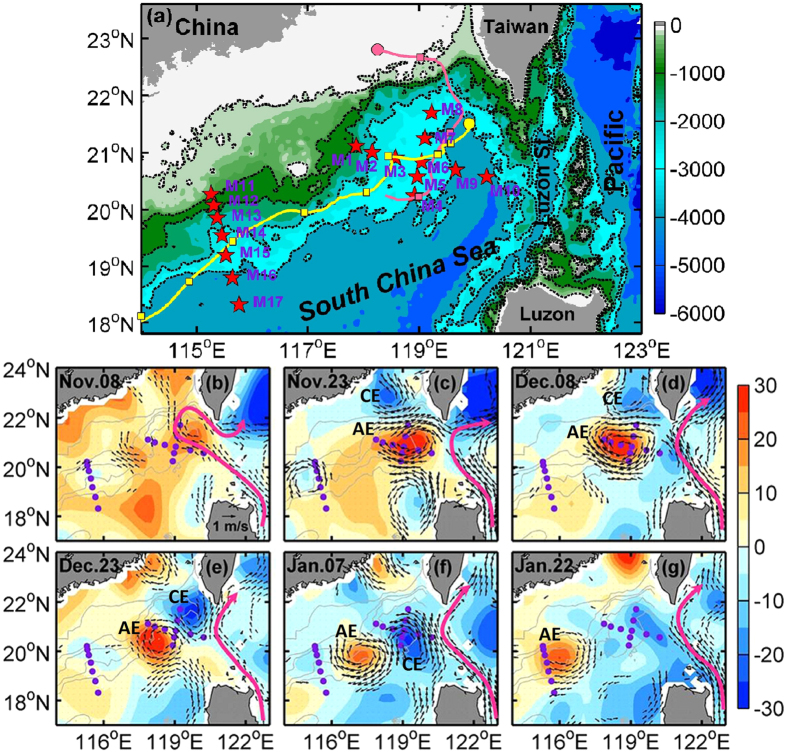
Observation of oceanic eddies. (**a**) Locations of the S-MEE mooring arrays (red stars). The color shading shows bathymetry with black dashed lines indicating the isobaths of 100, 500, 1000, 2000 and 3000 m, respectively. Yellow and magenta lines denote the trajectories of AE and CE in (**b-g**), respectively. The dot on the trajectory indicates the generation site of eddy, and the squares indicate eddy’s locations every 15 days after it was generated. Bathymetry data is downloaded from https://www.ngdc.noaa.gov/mgg/global/. (**b–g**) Maps of altimeter SLA (shading, in cm) and surface absolute geostrophic velocities (arrows, those with magnitudes smaller than 0.3 m/s are not shown) during the period of AE and CE (from 8 November 2013 to 22 January 2014). Purple dots indicate the mooring locations. Magenta line roughly denotes axes of the Kuroshio current. Locations of AE and CE are marked by ‘AE’ and ‘CE’, respectively, on the SLA maps. Altimeter data is distributed by AVISO (http://www.aviso.oceanobs.com). Figures are plotted using MATLAB R2013a (http://www.mathworks.com/). The maps in this figure are generated by MATLAB R2013a with M_Map (a mapping package, http://www.eos.ubc.ca/~rich/map.html).

To investigate the 3D structure and generation and dissipation processes of oceanic eddies in the SCS, we designed and conducted the S-MEE in the northern SCS. As part of S-MEE, two bottom-anchored subsurface mooring arrays were deployed along the historical pathway of mesoscale eddies in this region (Fig. 1a, Supplementary Fig. 1). The first array, cross-shaped and consisting of 10 moorings, was deployed west of the Luzon Strait in the generation region of eddies in late October 2013, and was successfully recovered in early June 2014. Before this experiment, mooring M1 at the western end of this array had been deployed and recovered two times, and it provided continuous measurements for a total length of more than 2 years. The second array, consisting of 7 moorings along a line normal to the local isobaths, was deployed in the dissipation region of eddies in mid-January 2013, and successfully recovered in mid-June 2014. All the moorings were equipped with acoustic Doppler current profilers (ADCPs), recording current meters (RCMs), conductivity-temperature-depths (CTDs) and temperature chains to monitor horizontal current velocity, pressure, and temperature/salinity (T/S) in the whole water column (see Methods and Supplementary Table S1). The moored velocity and T/S data are used to analyze the 3D structure and generation and dissipation processes of oceanic eddies.

In order to identify and track the oceanic eddies, AVISO merged satellite altimeter sea level anomaly (SLA) and absolute surface geostrophic velocity data during the experiment period (October 2013–June 2014) are used^34^. The temporal and spatial resolution of the AVISO data is daily and 1/4°, respectively. From the 9-month SLA and surface geostrophic velocity maps, a total of 5 distinct oceanic eddies were observed to cross the mooring arrays. Among these eddies, one anticyclonic and cyclonic eddy pair was fully captured by the first mooring array from mid-November to early January (Fig. 1b–e). After crossing the first array, the anticyclonic eddy (AE) propagated southwestward and was well measured by the second array from mid-January to early February (Fig. 1f,g). The AE during its two stages (at the first and second arrays) and the cyclonic eddy will be referred to as AE0, AE1 and CE, respectively, below. The analysis of this study will primarily focus on this eddy pair. On the basis of real-time SLA information, we also conducted two transects across the center of AE in early December 2013 and mid-January 2014, respectively (Supplementary Fig. 2). Along these two transects, high-resolution hydrographic and turbulent mixing measurements were made. The obtained turbulent kinetic energy dissipation rate (Supplementary Fig. 3a,b), CTD and shipboard ADCP velocity data are used to analyze the dissipation of AE.

### Generation of the eddy pair

**Figure 2:**
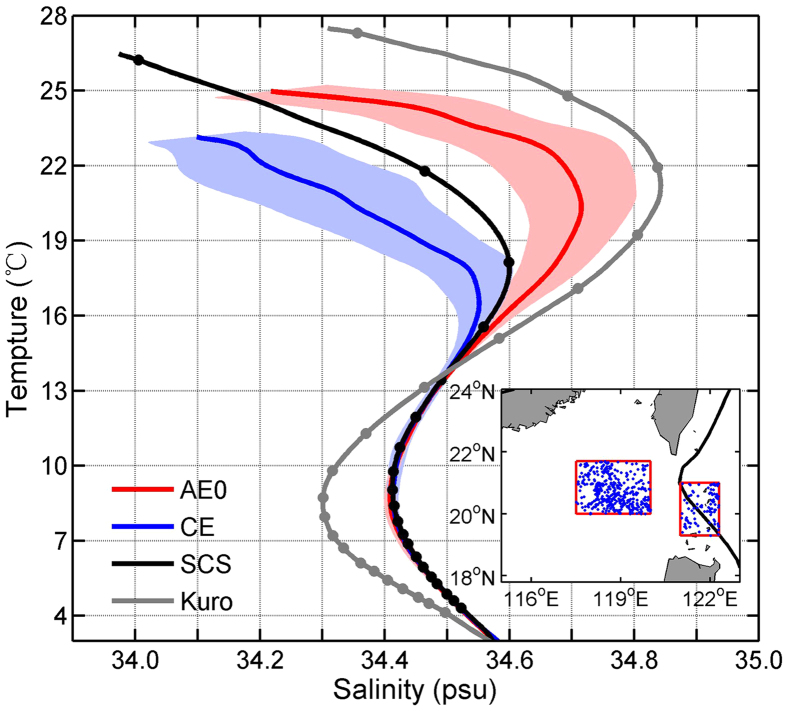
Mean T-S diagrams of water mass within and outside of the eddies. Red and blue lines show the results within AE0 and CE, respectively, obtained from the moored CTD observations (Supplementary Table S1). Shadings denote the standard deviations. The black and gray lines show the results of the northeastern SCS water and the Kuroshio water, respectively, computed based on historical Argo T/S profiles within the red boxes of the inset figure. The dots on the T-S diagrams indicate the depths of 50, 100, 150, 200 m, etc. Blue dots in the inset figure indicate Argo profiles’ locations, and the black curve indicates the axis of the time-mean Kuroshio. Figure is plotted using MATLAB R2013a (http://www.mathworks.com/). The map in the inset figure is generated by MATLAB R2013a with M_Map (a mapping package, http://www.eos.ubc.ca/~rich/map.html).

The AVISO SLA and geostrophic velocity fields reveal the generation of an anticyclonic and cyclonic eddy pair in the region southwest of Taiwan in the winter season of 2013/2014 (Fig. 1b–g). Before AE was generated, the Kuroshio, the western boundary current in the North Pacific subtropical gyre, appeared to intrude into the northeastern SCS in a form of loop current. After the generation of AE, the Kuroshio returned to its normal path, leaping across the Luzon Strait. This result suggests that the origin of AE is from the shedding of the Kuroshio current. For the CE, it was generated right behind the AE and seems to originate from the western part of the Taiwan Strait. The upper-layer (<350 m) water within the AE0 shows properties close to the Kuroshio water that is warmer and saltier than the SCS water (Fig. 2). The upper-layer water within the CE, on the other hand, is colder and fresher than the SCS water (Fig. 2). In addition, the high-resolution satellite sea surface temperature (SST) reveals a warm- and a cold-core within the AE and CE, respectively (Supplementary Fig. 4), and the historical hydrographic observations reveal a low temperature and salinity tongue in winter in the western Taiwan Strait^35^. All these results verified the AVISO observations that the AE was shed from the Kuroshio, while the CE originated from the western part of the Taiwan Strait which trapped cold and fresh near-shore water into the northern SCS. Based on 22 years (1993–2014) of the AVISO data, a total of 19 events of anticyclonic eddy shedding associated with the Kuroshio intrusion were detected. For more than 80% of these cases, a CE is immediately generated behind the AE to its northeastern side, suggesting the common occurrence of such eddy pairs (Supplementary Fig. 5). Formation of the trailing CE is likely due to the vortex tube stretching induced by the offshore transport on the northeast side of AE combined with the cyclonic velocity shear associated with the intruding Kuroshio south of Taiwan. Further study, however, is needed to verify this hypothesized formation mechanism for CE.

### 3D eddy structures

**Figure 3:**
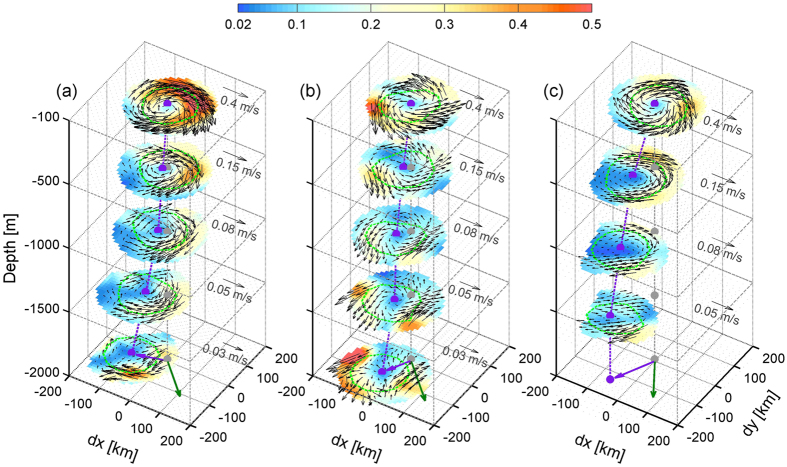
3D structure of the oceanic eddies. 3D structures of the horizontal velocity for (**a**) AE0, (**b**) CE and (**c**) AE1. Shading and black arrows denote the velocity magnitude and velocity vectors (in m/s), respectively. Note that velocity at different depths has different amplitude scales. The velocity amplitudes at 100, 500, 1000, 1500 and 2000 m have an amplifying ratio 1, 3, 5, 8 and 15, respectively. Green dotted line is the z-axis of the coordinate. Eddy centers at each layer, which are defined at the point of minimum velocity magnitude, are indicated by the purple dots. Edges of eddy core at each layer, which are defined at the radial positions of maximum velocity magnitude, are indicated by the green circles. Purple dotted line denotes the axis of eddy, defined as the line connecting the eddy centers at each layer. Purple arrow shows the drift of eddy center from the z-axis at the depth of 2000 m. Green arrow denotes the direction of the local topography gradient. Figures are plotted using MATLAB R2013a (http://www.mathworks.com/).

By combining the mooring-array and AVISO data, we constructed 3D structures of the eddies over the full water column (see Methods). Figure 3a–c show the 3D structures of horizontal velocity for AE0, CE and AE1, respectively. Vertically, the eddies are surface-intensified, with the velocity sharply decreasing with depth. At the same time, the eddies are found to be deep-penetrating, extending from the surface to near bottom (with depth over 2000 m). The maximum velocity magnitude at the 2000 m depth and deeper exceeds 3.5 cm/s and 5.0 cm/s for the AE0 and CE, respectively, which is much stronger than the time-mean current detected in the deep SCS circulation^36^,^37^. The deep signals of the eddy pair may be due to quick geostrophic adjustment in response to pressure anomalies caused by the upper-layer mass loading^38^. Horizontally, the velocity structure is axis-asymmetric and the velocity is enhanced on the northeastern side of AE0 and southwestern side of CE due to the lateral alignment of the eddy pair. The large current anomalies associated with the eddy pair are expected to play an important role in deep-ocean material transport as proposed by previous studies^32^,^39^,^40^.

Another notable feature for the 3D structures is that the axis of the eddies (purple dotted lines in Fig. 3) strongly tilts southwestward from surface to bottom. From the surface to 2000 m, the tilting distance reaches up to ~100 km for both AE0 and CE. For AE1, the tilting distance extends to ~150 km from the surface to 1500 m. These tilting structures of eddies can also be confirmed from the depth-time plots of velocity at each mooring (Supplementary Fig. 6e,f). Because the tilting direction is generally coincident with the propagation direction of the eddies (yellow and magenta lines in Fig. 1a), the phase of velocity in the deep layer is detected to lead that in the upper layer (Supplementary Fig. 6e,f). Quantitatively, the velocity phase at 2000 m leads that at 100 m by about 15 days, which, if multiplied by the eddies’ propagation speed of 0.08–0.11 m/s, agrees well with the aforementioned tilting distance. Although the reconstructed 3D thermohaline structures are confined to the upper 1000 m due to the limit of temperature chain data (Supplementary Table S1), tilting features similar to the velocity structures can also be clearly seen in them (Supplementary Fig. 7), i.e., eddy center tilts southwestward with increasing depth.

### Dissipation of the eddy

After its generation in early November, the AE quickly grew, and its amplitude (defined as the maximum SLA, see Methods) reached the maximum in early December (Fig. 1b–g). Then the AE propagated southwestward along the continental slope and its amplitude gradually decayed. The AE has a lifespan and travelling distance as long as 4.5 months and 900 km, respectively, and it finally disappeared north of the Xisha Islands (approximately at 112.5°E, 17.7°N) in mid March 2014. Compared to the AE, the CE is very short-lived and only has a lifespan of one and half months. After reaching its maximum amplitude in early January, the CE was quickly dissipated. Finally, it was observed to disappear just southwest of the first mooring array in mid January.

**Figure 4:**
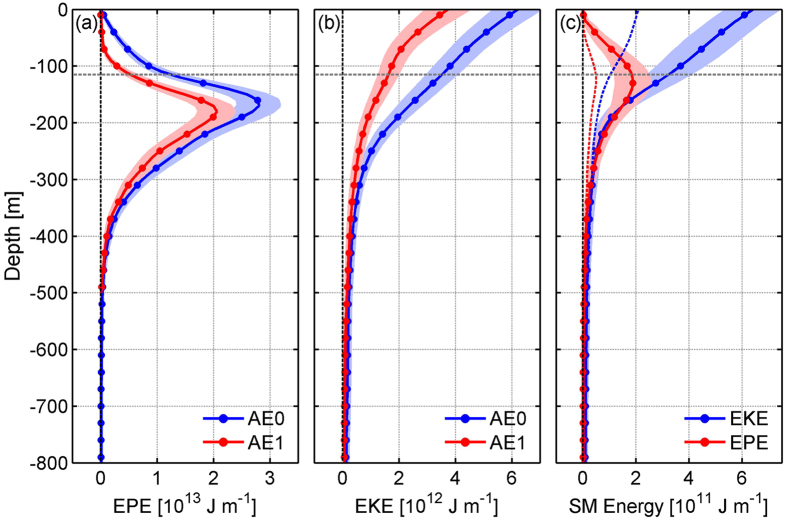
Vertical profiles of the eddy energy. (**a**,**b**) Area integrated (**a**) EPE_ms_ and (**b**) EKE_ms_ within AE0 (blue dotted line) and AE1 (red dotted line). Shadings denote the standard deviations. (**c**) Area integrated EKE_sm_ (blue dotted line) and EPE_sm_ (red dotted line) within AE. The result is an average of AE0 and AE1. Dashed lines denote the results outside of the eddies, with the blue and red ones denoting the EKE_sm_ and EPE_sm_, respectively. Gray dashed line in each figure denotes base of the mixed layer. Figures are plotted using MATLAB R2013a (http://www.mathworks.com/).

**Figure 5:**
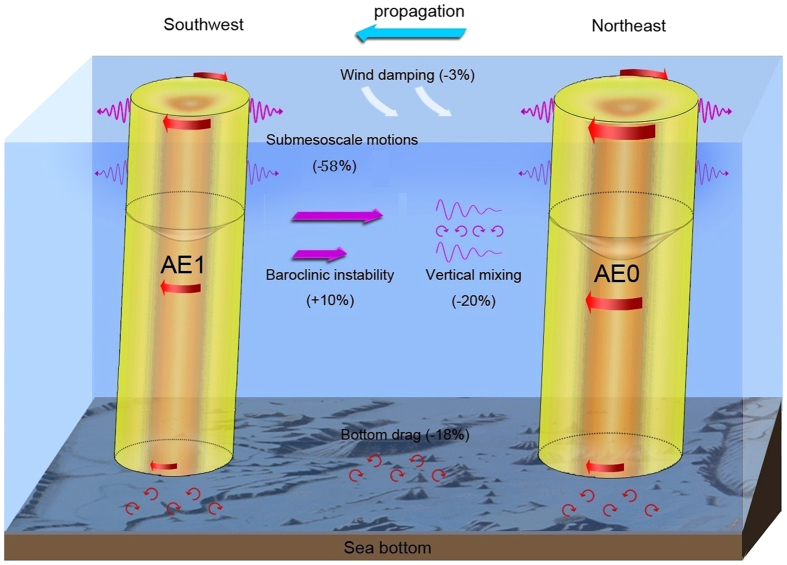
Schematic diagram of the eddy dissipation processes. Contribution of each dynamical process to the eddy dissipation is illustrated in this schematic. Bathymetry data used to draw the sea bed is from https://www.ngdc.noaa.gov/mgg/global/.

Because the AE was well captured by both of the mooring arrays, the eddy dissipation analysis hereafter is primarily focused on the AE. From AE0 to AE1, the AE’s amplitude, kinetic energy (EKE) and available potential energy (EPE) had a prominent decrease by 40%, 47% and 35%, respectively (Figs 1b–g and 4a,b). In order to investigate the dissipation mechanism of AE, we carried out an eddy energy budget analysis based on available observational data. The detailed quantification of each eddy energy budget terms is given in the Methods section. As summarized in Table 1 and schematically depicted in Fig. 5, summation of all the terms gives rise to a small residue (11% of the observed dissipated eddy energy (dEE) from AE0 to AE1), suggesting that the eddy energy budget is largely closed when considering the estimation uncertainties. During the decay process, the eddy-mean flow interaction still fed the AE by providing it eddy energy at the expense of the energy of mean circulation and accounted for 10% (±5%) of dEE. Both the surface wind stress and bottom drag did negative work on the AE (i.e., weakening the AE), but they accounted for only 3% (±2%) and 18% (±8%) of the dEE, respectively. Vertical eddy diffusion, with an observed vertical eddy diffusivity on order of 10^−4^ m^2^s^−1^ (Supplementary Fig. 3c,d; cf. background level is 10^−5^ m^2^s^−1^ in the ocean interior), explained 20% (±8%) of the AE’s dissipation. The enhanced vertical mixing is primarily caused by breaking of strong internal tides radiated from the Luzon Strait^41^,^42^ and it may also be partially due to the breaking of near-inertial waves radiating from the eddy itself^43^.

In the presence of AE, submesoscale motions (see definition in Methods) are prominently enhanced (Fig. 4c, Supplementary Fig. 8a), with the submesoscale eddy energy within the AE more than double that outside of the AE (Fig. 4c). The close correlation between the submesoscale EKE (KE_sm_) and mesoscale EKE (KE_ms_) is confirmed by examining the 2-year moored observations (Supplementary Fig. 9), implying that the mesoscale eddies can feed the generation and growth of submesoscale motions. If we assume that the increased submesoscale energy within the AE all derives from AE, this part of energy would account for 58% (±12%) of the dEE over the period from AE0 to AE1 (see the detailed calculation in Methods). This implies that the generation of submesoscale motions, or downscale energy transfer, likely plays a dominant role in the dissipation of AE.

Taking into account that the submesoscale motions act on horizontal velocity shear of AE, this amount of dissipated energy (*D*_*h*_·*dt* in Table 1) yields an “effective horizontal eddy viscosity” (i.e., *A*_*h*_) of 75–110 m^2^s^−1^ for submesoscale motions (see Methods), which is within the range of previous estimates based on observations and numerical simulations^28^,^44^,^45^. The reasonable *A*_*h*_ value estimated here in turn supports the notion that the downscale energy cascade/transfer is likely the primary dissipation mechanism for AE.

## Discussion

Based on observations from individual moorings in the northern SCS, previous studies found that, during the eddy pair events the deep current (>2000 m) was intensified and tended to flow in opposite directions against the surface current^32^,^40^. The deep-penetrating and vertical-tilting eddy structure found in this study provides a good explanation for this previously observed phenomenon. Because the eddy propagation in the northern SCS follows the regional sloping bottom topography, the topographic β effect, which exerts more influence to the water column near bottom in the stratified ocean^46^, is likely the cause for the observed vertically tilting structures. Indeed, previous modelling studies showed that for an isolated equivalent barotropic eddy overriding a sloping topography, the topographic β effect works to shift the eddy core in the lower layer more to the right in the down-slope direction than in the upper layer^47^. It is important to emphasize that the mesoscale eddies reconstructed in the open ocean exhibited no such strongly tilted 3D structures^4^,^8^,^9^,^10^ and, as such, the observed tilting structure is likely a unique characteristic of eddies in the northern SCS or other marginal seas with broad-scale continental slopes.

In defining submesoscale motions in the present study, we have adopted the separation method based on time scales (see Methods). It is worth pointing out that the submesoscale motions thus derived show characteristics consistent with the classical submesoscale dynamics. Specifically, different from the quasi-geostrophic mesoscale eddies whose EPE_ms_ is larger than EKE_ms_ (Fig. 4a,b), EKE_sm_ is comparable with EPE_sm_ for the submesoscale motions (Fig. 4c). This results in an order one Rossby number 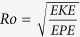 38, highlighting the ageostrophic feature of the submesoscale motions. Compared to the mesoscale eddies, submesoscale energy decreases more rapidly with depth and is primarily concentrated within the surface mixed layer (Fig. 4, Supplementary Fig. 10). All these characteristics indicate that the submesoscale motions identified in this study fall in the classical submesoscale regime^26^,^48^,^49^.

In addition to the perspective based on the time scale separation, submesoscale motions can also be seen from a spatial scale perspective. High-resolution (1 km) SST images reveal fine submesoscale structures (of horizontal scales under 50 km) along the peripheries of the cores of AE and CE (Supplementary Fig. 4). From the high-resolution (1 km) shipboard velocity measurements (Supplementary Fig. 2c,d) we also found that the submesoscale motions with horizontal scales smaller than 50 km show enhanced EKE_sm_ on the peripheries of the AE’s core. In addition, the high-resolution velocity data produced an energy spectrum with a spectral slope of k^−2^ (k is the horizontal wavenumber), which characterizes the submesoscale regime^26^,^50^. Based on these results, the timescale-based submesoscale motions defined in this study show no substantial difference with those defined based on spatial scales. Although the eddy energy budget analysis was applied to only the AE case, enhanced submesoscale energy was also found in other mesoscale eddy cases based on the 2-year moored observations at M1 (Supplementary Fig. 9). Based on the above observational evidence, we conclude that the downscale energy transfer to submesoscale motions likely plays an important role for the dissipation of oceanic eddies in the northern SCS. Finally, we note that the downscale energy cascade derived in this study is for the total eddy energy (EKE + EPE) signals. An inverse energy cascade is likely to occur if the EKE only is considered.

## Methods

### *In-situ* moored data

To acquire the full-depth current velocity and T/S data, self-contained instruments including ADCPs, RCMs, CTDs and temperature chains were heavily mounted on the moorings (Supplementary Fig. 11 and Table S1). The instrument configuration on each mooring is similar but not identical due to the different water depth at the mooring locations. Nominally, the moorings were equipped with an upward-looking and a downward-looking 75 kHz ADCPs (sometimes only one upward-looking ADCP) at ~500 m to measure the velocity in the upper 1000 m (or 500 m) water column. Below that, discrete RCMs provided point measurements of velocity every 400–600 meters, with the upmost one at the nominal depth of 1500 m (or 550 m) and the lowermost one at depth 50–200 meters above the ocean bottom. Immediately beneath each RCM (for most cases), a CTD was mounted on the mooring to measure the T/S and pressure. The moorings were also equipped with temperature chains extending from tens meters beneath the sea surface to ~1000 m (or ~500 m), consisting of 2–6 CTDs and dozens of thermometers. Detailed mooring configurations can be found in Table S1 of the supplementary information.

The high-resolution raw velocity data were firstly hourly averaged. For each mooring, the hourly velocity data from both ADCPs and RCMs were then linearly interpolated onto the uniform 10 m levels between near surface (40–60 m) and near bottom. In order to remove the major tidal and inertial signals (the inertial period is 31–38 hours in the study region), all the hourly velocity time series were 2-day low-pass filtered with a fourth-order Butterworth filter. The low-pass filtered velocity data were further averaged on a daily basis. The processing procedure as described above was similarly applied to the temperature chain data, resulting in the daily-averaged temperature data at 10 m depths between the near surface and ~1000 m (or ~500 m). The aforementioned daily velocity and temperature data are used for analysis in this study.

### Mesoscale and submesoscale motions

The observed temperature and velocity time series show strong intraseasonal variations with periods between 2 and 100 days (Supplementary Fig. 8). The intraseasonal variations are associated not only with the mesoscale eddies, but also other high-frequency motions embedded in the eddies (Supplementary Fig. 8a). The periods of these high-frequency motions are generally shorter than 10 days, which can be clearly seen from the power spectra (Supplementary Fig. 8). To be distinguished from the oceanic eddies, the high-frequency motions with periods shorter than 10 days are regarded as submesoscale motions in this study. The temperature anomaly (*T*′) on the intraseasonal timescale is similarly separated into two terms, 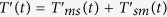, where 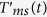 and 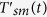 denote the 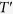
relating to the mesoscale and submesoscale motions, respectively. Here, we derive 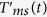 using a 10-day low-pass filter and 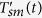 using a 10-day high-pass filter. The same separation was also applied to the velocity data. Throughout the paper, the subscripts ‘ms’ and ‘sm’ are used to denote variables associated with the mesoscale and submesoscale motions, respectively.

### Construct the 3D structures

The moored and altimeter data are combined to construct the 3D structures of the oceanic eddies (AE0, CE and AE1). Before the construction of eddy’s 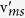
and 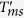 field, the Okubo-Weiss parameter was first used to identify the eddies from the SLA maps^51^,^52^. The criterion of *W* < −0.2*σ*_*w*_ is used to define the eddy element within the eddy core, where *W* is the Okubo-Weiss parameter and 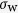
is the spatial standard deviation of *W* in the study region in each day. *W* is defined as 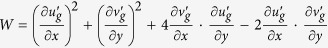, where 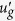
and 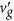
are geostrophic velocity anomalies calculated from the SLA through geostrophy. We use the mean longitude and latitude of the eddy elements to represent the eddy center (*x*_*0*_, *y*_*0*_), and the radius of an equal-area circle to represent the eddy radius *R*_*0*_. We define the amplitude of the eddy (*H*_*0*_) as the maximum SLA around the eddy center. With the above altimeter-derived surface information, we tracked the eddies based on a previously used eddy tracking algorithm^30^. Then, we derived the trajectory (Fig. 1a) and temporal evolution of each oceanic eddy.

For eddy AE0, its shape was relatively steady from 14 November to 27 December 2013. Based on the moored data during this period, the 3D structure of AE0 was constructed as follows. Taking the AE0 center as the origin, we constructed a new cartesian coordinate in each day. Then, all the 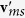 and 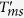 profiles located within 200 km of the eddy center were projected onto this new coordinate system. Considering the variation of the eddy size during the given period, we normalized the coordinates (*x, y*) of the profiles using 
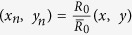
, where (*x*_*n*_*, y*_*n*_) denote the normalized coordinates, and *R*_0_ and 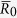 are the instantaneous and mean eddy radius, respectively. 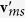
and 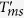
were normalized by *H*_*0*_ of the eddy with the similar method. At each depth, the obtained 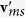
and 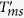 were then mapped onto a 10 km by 10 km grid using an objective interpolation scheme. The weight function for the mapping has an inverse distance form, 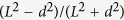, where *L* = 50 km is the interpolation radius, and *d* is the distance between the data point and grid point. For each grid point, only the data points with *d* < *L* are used. Finally, the 3D structure of 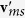 in the full water column and 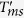 in the upper 1000 m were obtained for AE0. Using the data from 14 December 2013 to 8 January 2014 (from 14 January to 10 February 2014), the 3D structure of CE (AE1) was also constructed based on the same method. The similar method to construct eddy’s 3D structure was also used by the previous studies^4^,^8^,^9^,^10^. The difference is that the previous studies reconstructed the mean structure of many eddies because there were very few observations for individual eddies. To get the mean structure, those studies had to assume that all eddies have similar 3D structures.

### Eddy energy budget

From the primitive equations for Boussinesq fluid we derived the eddy kinetic energy (EKE) and eddy available potential energy (EPE) equations^53^,^54^. Adding the EKE and EPE equations and integrating over the whole water column, we obtained


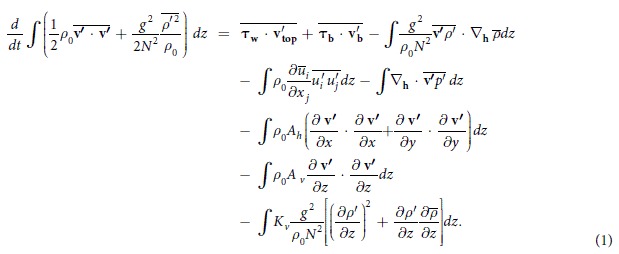


Here, primes denote the mesoscale anomalies; overbars denote the time average; 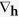 is the horizontal gradient operator; 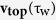 is the horizontal velocity (wind stress) at the sea surface; 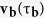 is the horizontal velocity (drag stress) near the sea bottom; 
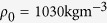
 is the mean sea water density; *N* is the buoyancy frequency; *A*_*h*_, *A*_*v*_ and *K*_*v*_ denote the horizontal eddy viscosity, vertical eddy viscosity and vertical eddy diffusivity, respectively; the other symbols and notations are standard. The above equation can be written symbolically as


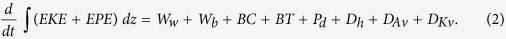


The rhs terms, respectively, are rate of the wind stress work (*W*_*w*_) and the bottom drag work (*W*_*b*_), baroclinic (*BC*) and barotropic (*BT*) conversion terms of the eddy-mean flow interaction (if positive, the mean flow transfers energy to the eddy), divergence of perturbative pressure work (*P*_*d*_), and terms of energy dissipation caused by horizontal eddy viscosity (*D*_*h*_), vertical eddy viscosity (*D*_*Av*_) and vertical eddy diffusivity (*D*_*Kv*_). The last two terms (*D*_*Av*_ and *D*_*Kv*_) are termed *D*_*v*_ for simplicity.

AE was observed two times by the mooring arrays, one during its mature stage (i.e. AE0) and the other during its decay stage (i.e. AE1). The energy budget of AE, therefore, can be analyzed in a Lagrangian framework. Using the formulas in equation (1), we first calculated EKE and EPE of AE0 (AE1) based on its 3D structure of 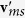 and 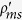, respectively. The 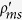 here was calculated based on 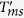 assuming that contribution of salinity anomaly to 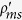 is negligible^32^. The eddy energy (EE, i.e. EKE + EPE) was then integrated over the eddy: vertically, from 500 m to the surface; horizontally, from the center to the edge of the eddy. Here, the eddy center at each depth is defined as the point with the minimum EKE or maximum EPE; the eddy edge here is defined as the points where EE is 5% of its maximum value. The 500 m was chosen as the lower limit of integration because the temperature observations below this depth are very scarce for AE1 (Supplementary Table S1). For AE0, the integrated EE over the upper 500 m accounts for more than 95% of the total EE, and as such, we believe that this choice does not affect the conclusions. In the Lagrangian framework, EE of AE1 minus that of AE0 equals to time integral of the rhs terms. Given that there were no observations between the two mooring arrays, we assumed that the rhs terms had a linear evolution from AE0 to AE1. As a result, time integral of the rhs terms equals to the averaged values of AE0 and AE1 multiplied by the time for AE to move from AE0 to AE1 (~60 days).

To quantify the rhs terms of AE0 and AE1, we utilized their 3D structures constructed using the available data, and integrated them over the eddy entity. In the calculation


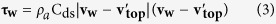


55where 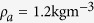 is the density of air at sea surface, C_ds_ is the drag coefficient as a function of wind speed^56^, 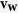 is the 10-m wind velocity of the ASCAT wind product^57^, and 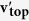 is the mesoscale sea surface velocity derived from the altimeter data. The bottom drag stress 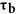 is formulated as


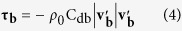


14where C_db_ is a constant bottom drag coefficient (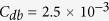), and 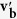 is the mesoscale velocity measured by the deepest RCMs (50–200 m above the sea floor). The horizontal density gradient in the BC term is approximated by the thermal wind relation


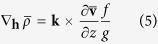


where 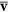 is the mean velocity. In the BT term, there exists horizontal gradient of mean velocity, which cannot be determined by the available data except at station M6 (using centered difference). The calculated BT at M6 was one order of magnitude smaller than the BC term and it therefore was assumed to be negligible. Because the streamlines of geostrophic oceanic eddies are generally along the P′ (pressure anomaly) contours, the P_d_ term is commonly small and will also be neglected. In the quantification of D_v_ term, the mean profiles of *A*_*v*_ and *K*_*v*_ calculated based on the observed turbulent kinetic energy dissipation rate (Supplementary Fig. 3) were used^58^.

Because of the unknown parameter *A*_*h*_, the *D*_*h*_ term cannot be directly calculated. Here, we estimated this term in an indirect way as below. Physically, *D*_*h*_ is the energy dissipation caused by submesoscale motions that act on horizontal velocity shear of mesoscale eddies, and as a result, it represents the energy mesoscale eddies transfer downscale to the submesoscale motions. Therefore, this term can be estimated if we know how much submesoscale energy was generated by the AE over the period from AE0 to AE1. As seen from Fig. 4c (also Supplementary Figs 8a and 9), in the presence of AE, submesoscale motions are prominently enhanced and the submesoscale eddy energy within the AE is more than double that outside of the AE. Based on the instability theory of the Eady model^50^,^59^, the observed mixed-layer mean stratification (N = 3.4 × 10^−3^ s^−1^) and geostrophic shear (Λ = 1.2 × 10^−3^ s^−1^) within and around the AE give rise to a mean growth rate (0.3*f*Λ/*N*, f is the local inertial frequency) of 1/2.2 day^−1^ for the most unstable mode of submesoscale motions in the mixed layer. If we assume that the increased submesoscale energy within the AE all derives from AE and that the submesoscale motions have a mean growth rate of 1/2.2 day^−1^, it would result in a total of 11.5 × 10^14^ J eddy energy transferring downscale from the AE to the submesoscale motions over the period from AE0 to AE1 (~60 days). Here, the calculation formula is 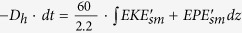
(6), where 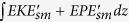 denotes the increased submesoscale energy within the AE (solid lines minus dashed lines in Fig. 4c, and integrated over the depth) and dt is 60 days. Based on this *D*_*h*_ value, the horizontal eddy viscosity can be estimated with 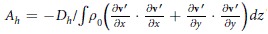
(7), where 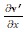 and 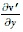 are the horizontal velocity shears of AE.

## Additional Information

**How to cite this article**: Zhang, Z. *et al.* Observed 3D Structure, Generation, and Dissipation of Oceanic Mesoscale Eddies in the South China Sea. *Sci. Rep.* **6**, 24349; doi: 10.1038/srep24349 (2016).

## Supplementary Material

Supplementary Information

## Figures and Tables

**Table 1:** Terms contributing to the dissipation of AE.

	dEE	BC·dt	W_w_·dt	W_b_·dt	D_v_·dt	D_h_·dt	Resi
Energy (10^14^ J)	−19.7	1.9 ± 1.1	−0.6 ± 0.5	−3.5 ± 1.5	−4.0 ± 1.6	−11.5 ± 2.3	−1.4
Contribution (%)	−100	10 ± 5	−3 ± 2	−18 ± 8	−20 ± 8	−58 ± 12	−11

All energy values are volume-integrated over the AE. Contribution is the ratio of each energy term to dEE. Resi denotes the residue of the eddy energy budget. See detailed definitions and calculation of each term in Methods. Values to the right of the ± symbol denote the uncertainty of each term. The uncertainty is primarily associated with the fact that there were no *in-situ* observations between the first and second mooring arrays and we assumed a linear evolution for AE between them (from AE0 to AE1). Here, it is estimated as the half value of the difference between that we integrated the observed terms at AE0 and AE1, respectively, during the whole period.
