# Supplementary material for: Observed 3D Structure, Generation, and Dissipation of Oceanic Mesoscale Eddies in the South China Sea
Source: Sci Rep. 2016 Apr 14;6:24349. doi: 10.1038/srep24349 (PMC4830941; doi:10.1038/srep24349)
Supplement: Supplementary Information [file srep24349-s1.doc]

**Supplementary Information**

**Observed 3D Structure, Generation, and Dissipation of Oceanic Mesoscale Eddies in the South China Sea**

Zhiwei Zhang^1^, Jiwei Tian^1,*^, Bo Qiu^1,2^, Wei Zhao^1^, Ping Chang^1,3^, Dexing Wu^1^, Xiuquan Wan^1^

Physical Oceanography Laboratory/Qingdao Collaborative Innovation Center of Marine Science and Technology, Ocean University of China, 238 Songling Road, Qingdao 266100, P.R. China.

^2^Department of Oceanography, University of Hawaii at Manoa, 1000 Pope Road, Honolulu, Hawaii 96822, USA

^3^Department of Oceanography, Texas A&M University, College Station, Texas 77843, USA

^*^**Corresponding author:** Jiwei Tian (Physical Oceanography Laboratory/Qingdao Collaborative Innovation Center of Marine Science and Technology, Ocean University of China, 238 Songling Road, Qingdao 266100, P.R. China; Telephone: +86-0532-66782367; Email: tianjw@ouc.edu.cn)


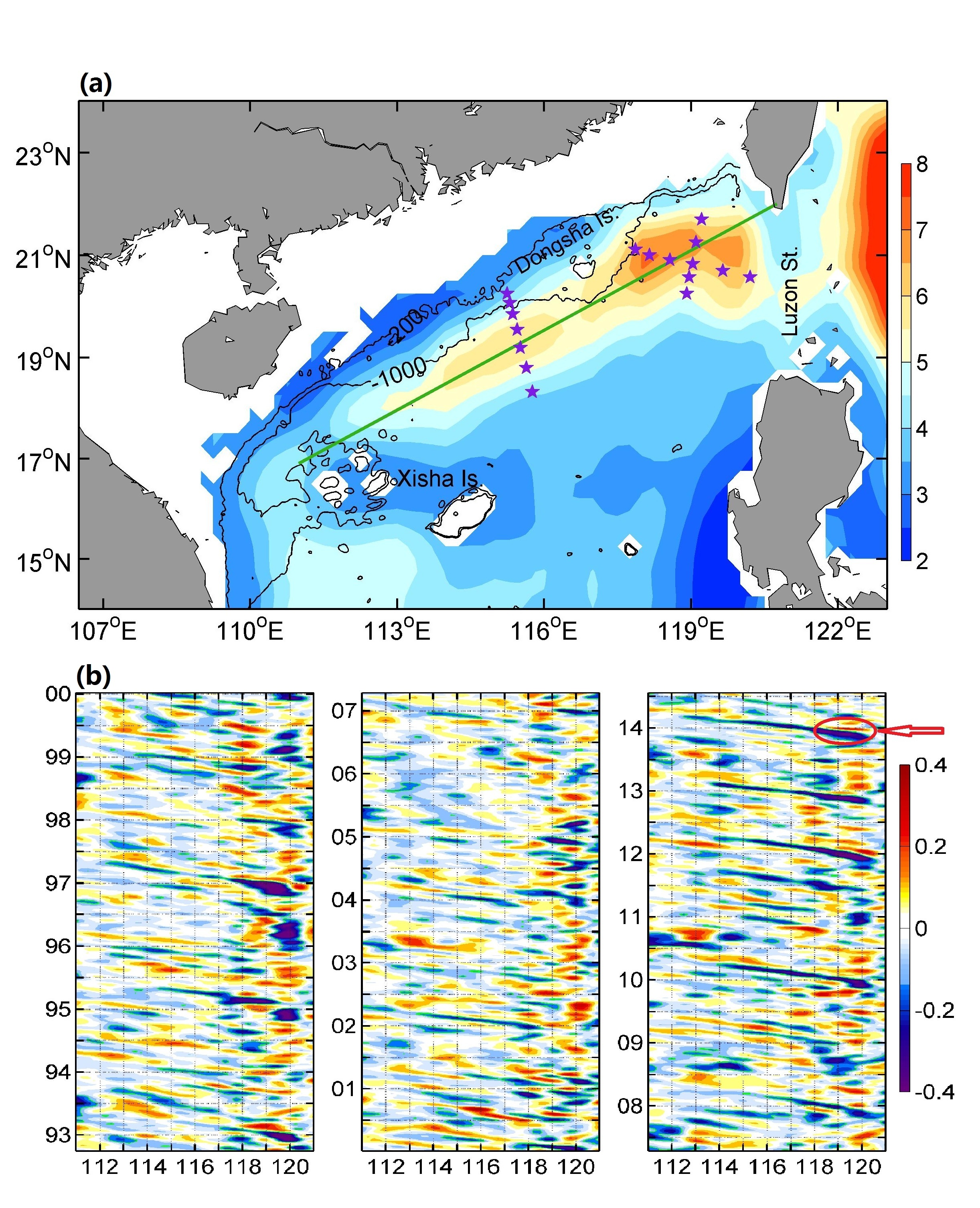


**Supplementary Figure S1 | Eddy activities in the northern SCS.** **(a)** Root-mean-squared SLA variability (in cm) in the northern SCS based on 140-day high-pass-filtered altimeter data from October 1992 to June 2014. The mooring locations are indicated by the purple stars. **(b)** Time-longitude plots of the surface relative vorticity ($\zeta$) along the green line in Fig.S1a. The $\zeta$is calculated based on the altimeter SLA and is normalized by the local planetary vorticity. The green contours represent $\zeta$value of -0.1. The horizontal and vertical axis denote the longitude and time (year), respectively. The eddy pair studied in this paper is indicated by the red ellipse and arrow. Figures are plotted using MATLAB R2013a (http://www.mathworks.com/). The map in this figure is generated by MATLAB R2013a with M_Map (a mapping package, http://www.eos.ubc.ca/~rich/map.html).


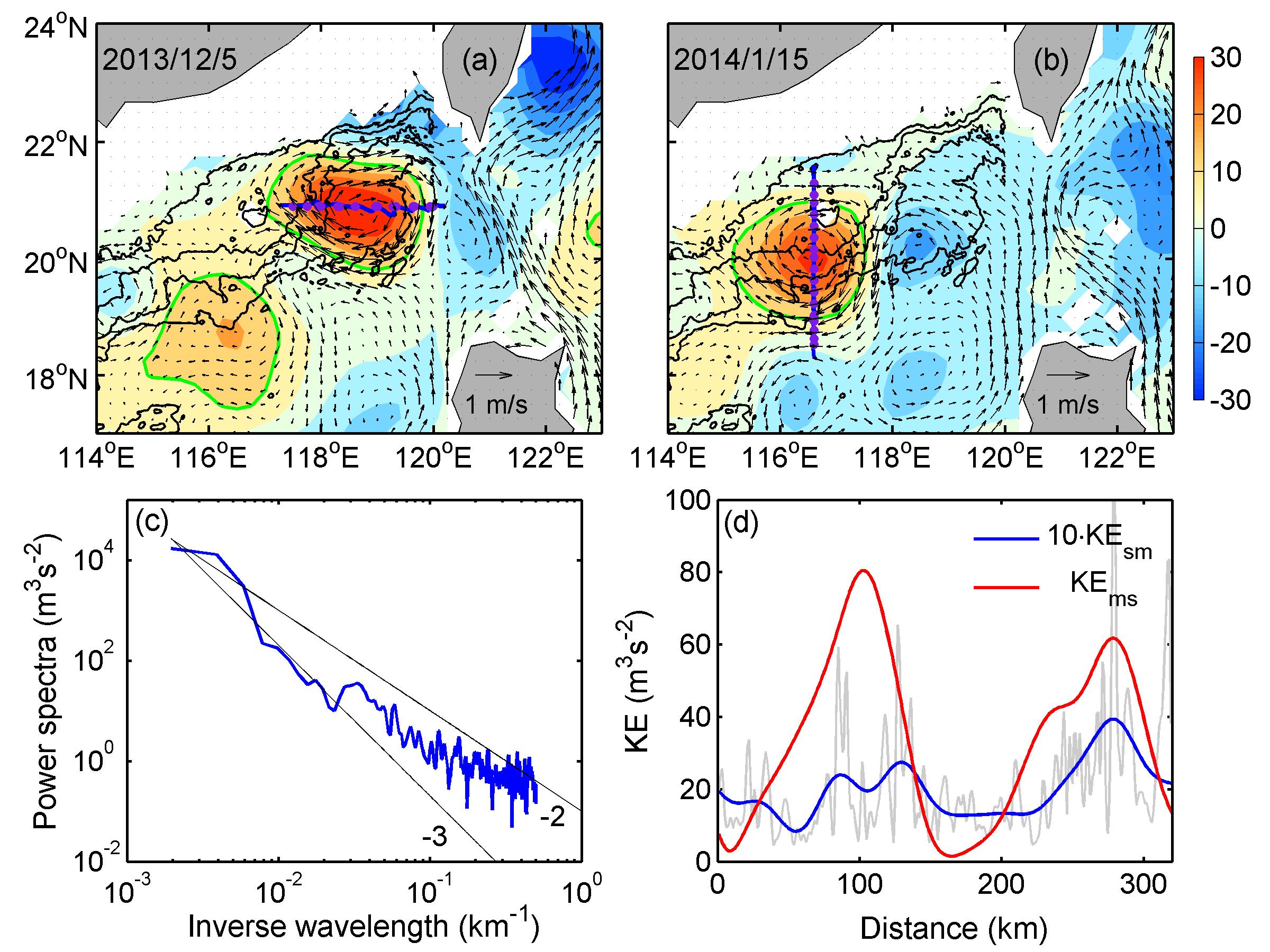


**Supplementary Figure S2 | Locations and results of the shipboard measurements.** Locations of the hydrographic and turbulence mixing measurement (purple dots) made **(a)** between 4 and 5 December 2013 and **(b)** between 14 and 16 January 2014. Blue line denotes the ship track of shipboard ADCP measurements. The color shading shows the altimeter SLA (in cm) and the black arrows indicate the surface geostrophic velocities (in m/s). Regions shallower than 200 m are masked by white shading. **(c)** Kinetic energy spectrum at a depth of 45 m (blue line) calculated based on the shipboard ADCP velocities along the blue line in **(a)** with a horizontal resolution of 1 km. The two black lines indicate the slope of -2 and -3, respectively. **(d)** Depth-integrated (over the upper 500 m) KE_ms_ (red line) and tenfold KE_sm_ (gray line) as a function of the integral distance from the eastmost point of the ADCP measurements along the blue line in **(a)**. The mesoscale and submesoscale velocities are obtained using a 50 km low-pass and high-pass filter, respectively. Blue line is the 50 km low-pass filtered KE_sm_. Figures are plotted using MATLAB R2013a (http://www.mathworks.com/). The maps in this figure are generated by MATLAB R2013a with M_Map (a mapping package, http://www.eos.ubc.ca/~rich/map.html).


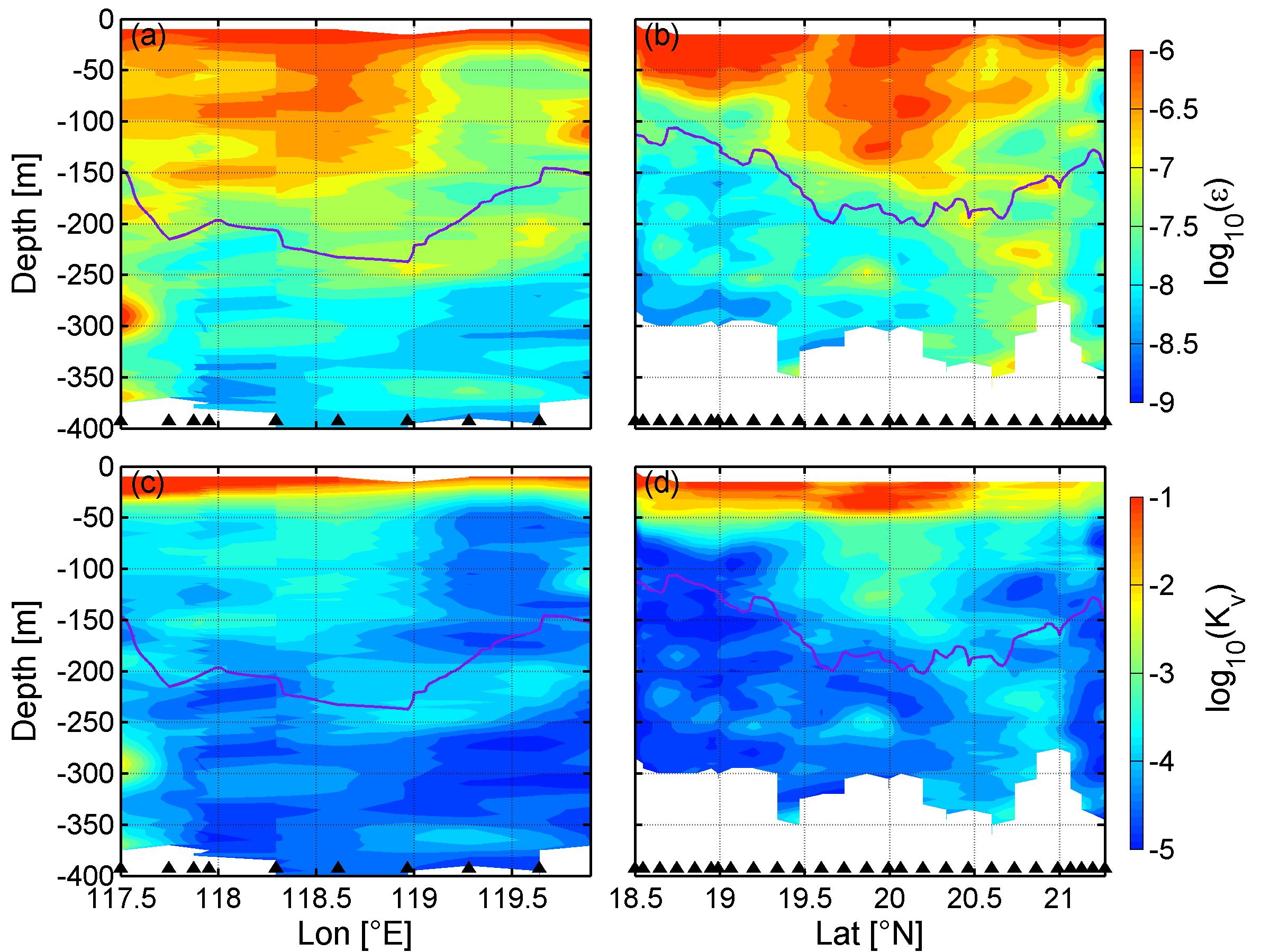


**Supplementary Figure S3 | Observed turbulent dissipation rate and vertical eddy diffusivity.** Observed turbulent kinetic energy dissipation rate ($\varepsilon$, unit in m^2^s^-3^) along **(a)** the zonal section in Fig. S2a and **(b)** the meridional section in Fig. S2b. Color bar of the dissipation rate is logarithmic. Purple line is the 20 ℃ isotherm, roughly indicating the location of thermocline. Black triangles at the bottom indicate locations of observation sites. Figures **(c, d)** are the same with figures **(a, b)**, respectively, but for the vertical eddy diffusivity (*K_v_*, unit in m^2^s^-1^). Figures are plotted using MATLAB R2013a (http://www.mathworks.com/).


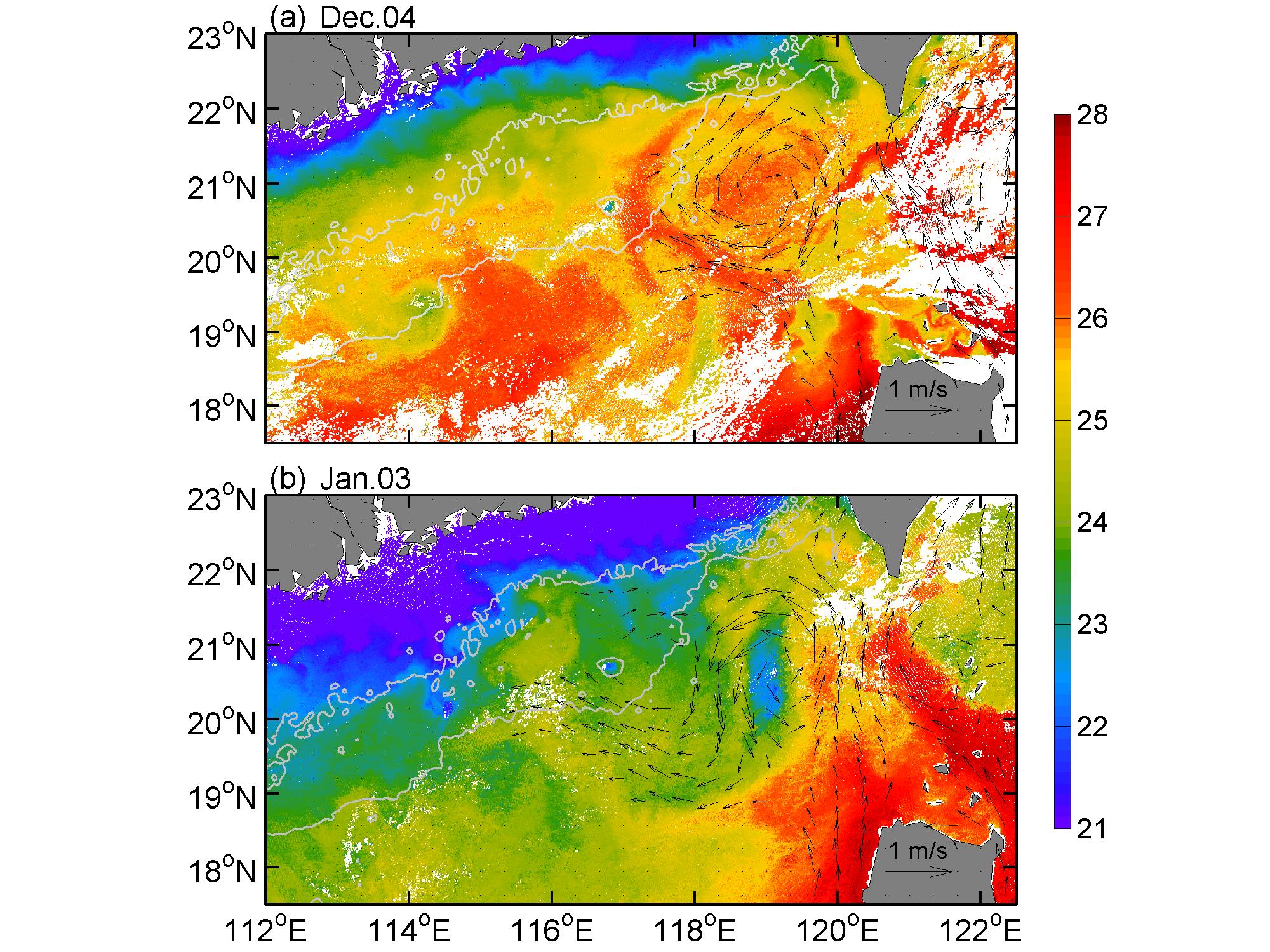


**Supplementary Figure S4 | The maps of high-resolution (1 km) sea surface temperature (SST).** The SST (in ℃) composite from Level-2 MODIS Aqua and Terra SST **(a)** between 3 and 5 December 2013 and **(b)** between 2 and 4 January 2014 (data downloaded from http://oceancolor.gsfc.nasa.gov/). Missing data are indicated by white shading. The black vectors are the altimeter-derived surface geostrophic velocity associated with the AE0, CE and Kuroshio. The gray lines denote the 100 and 1000 m isobath. Figures are plotted using MATLAB R2013a (http://www.mathworks.com/). The maps in this figure are generated by MATLAB R2013a with M_Map (a mapping package, http://www.eos.ubc.ca/~rich/map.html).


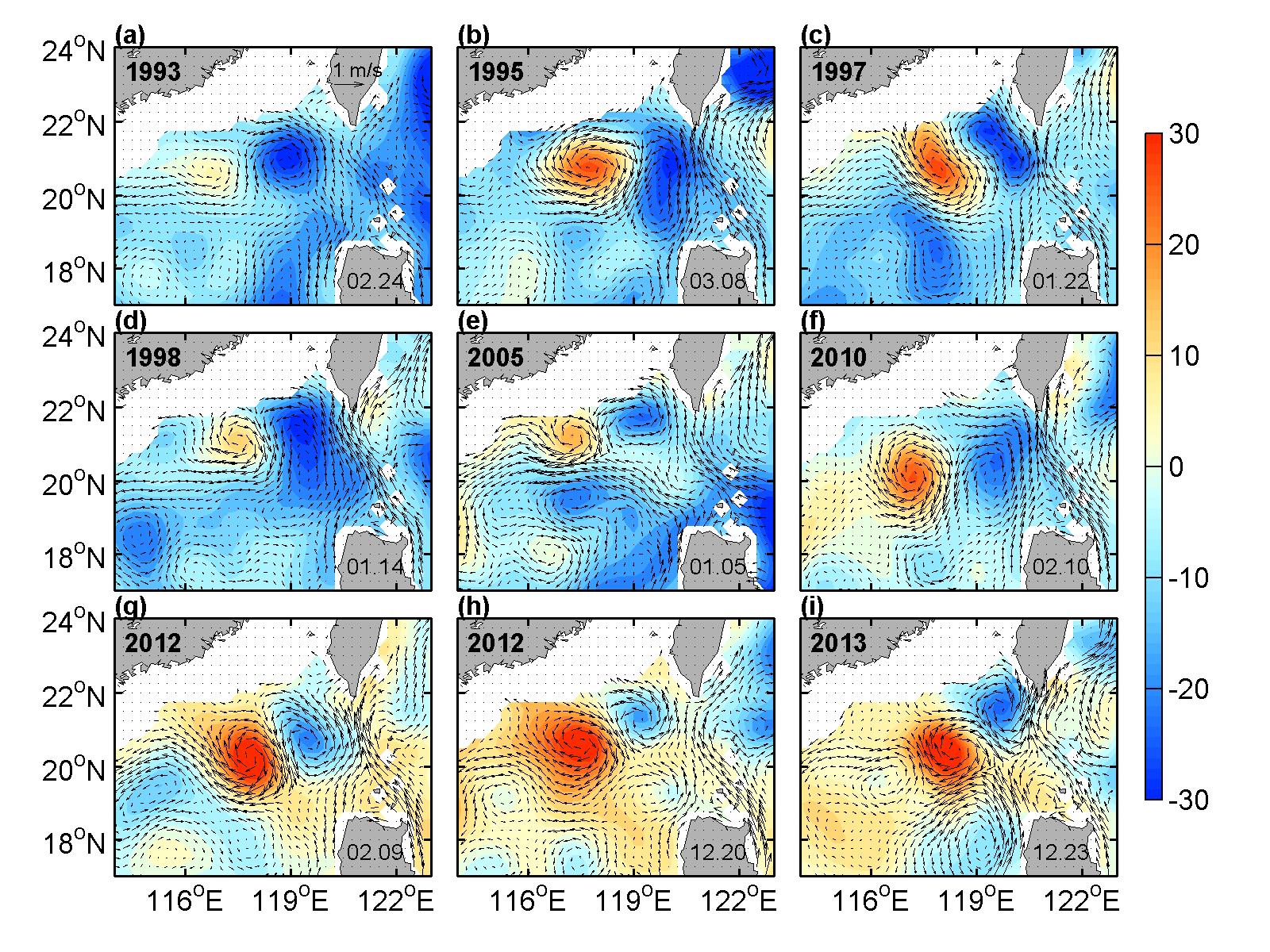


**Supplementary Figure S5 | Examples of Oceanic eddy pairs in different years.** Maps of altimeter SLA (color shading, in cm) and surface geostrophic velocities (black arrows, in m/s) on **(a)** 24 Feb 1993, **(b)** 8 Mar 1995, **(c)** 22 Jan 1997, **(d)** 14 Jan 1998, **(e)** 5 Jan 2005, **(f)** 10 Feb 2010, **(g)** 9 Feb 2012, **(h)** 20 Dec 2012 and **(i)** 23 Dec 2013. Regions shallower than 200 m are masked by white shading. Figures are plotted using MATLAB R2013a (http://www.mathworks.com/). The maps in this figure are generated by MATLAB R2013a with M_Map (a mapping package, http://www.eos.ubc.ca/~rich/map.html).


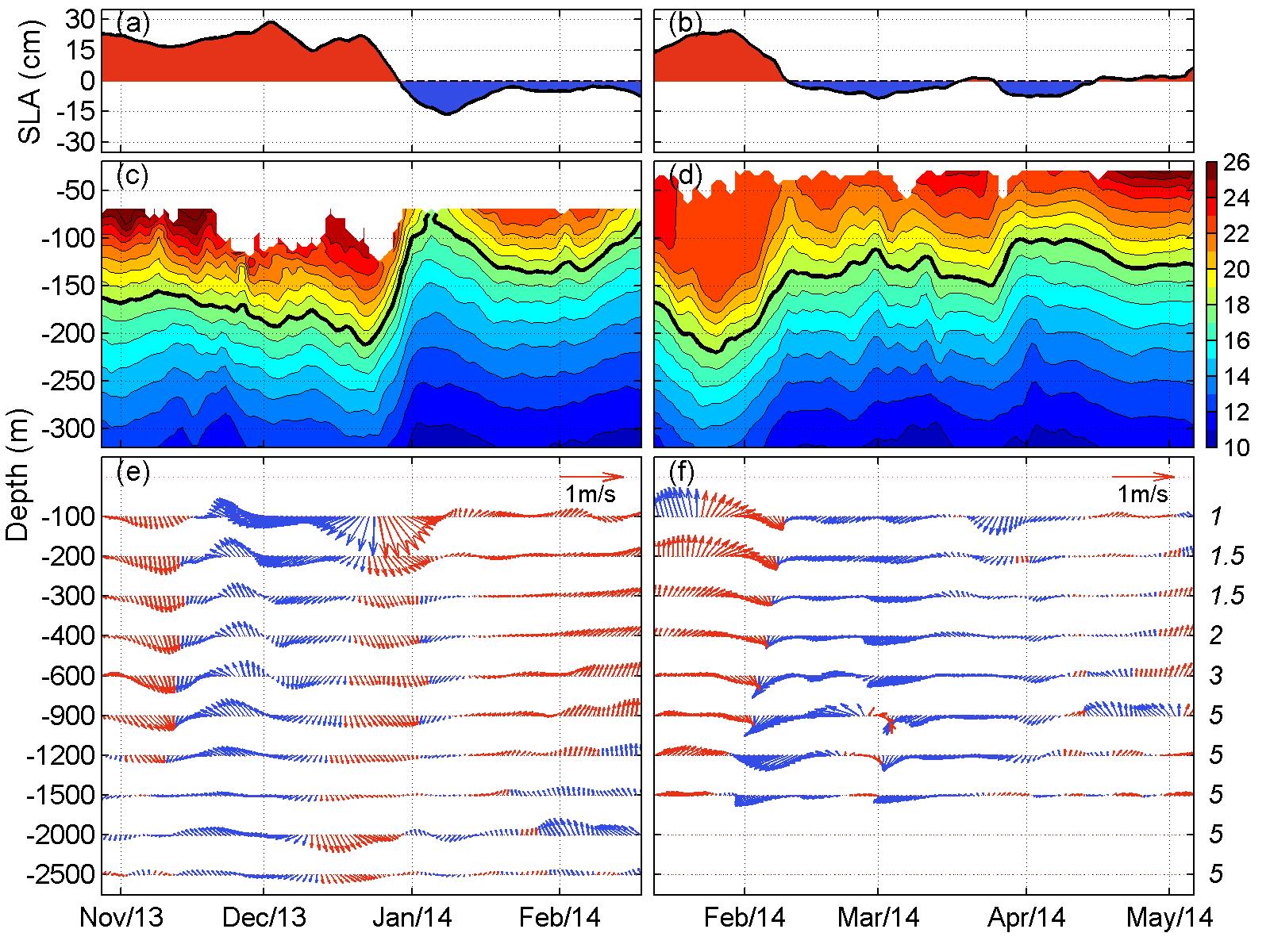


**Supplementary Figure S6 | Time series of the observations.** Time series of the observed **(a)** SLA, **(c)** temperature (in ℃) and **(e)** velocity vectors at mooring site M4. Figures **(b, d, f)** are the same with figures **(a, c, e)**, respectively, but for the mooring site M13. In figures **(a, b)** the positive and negative values are indicated by red and blue shadings, respectively. In figures **(c, d)** the contour interval is 1℃ and the 18 ℃ isotherm is indicated by the thick black line. In figures **(e, f)** velocity vectors with an eastward (westward) component are plotted in red (blue) color. The velocity magnitude at each layer is multiplied by a ratio marked on the right of **(f)**. Figures are plotted using MATLAB R2013a (http://www.mathworks.com/).


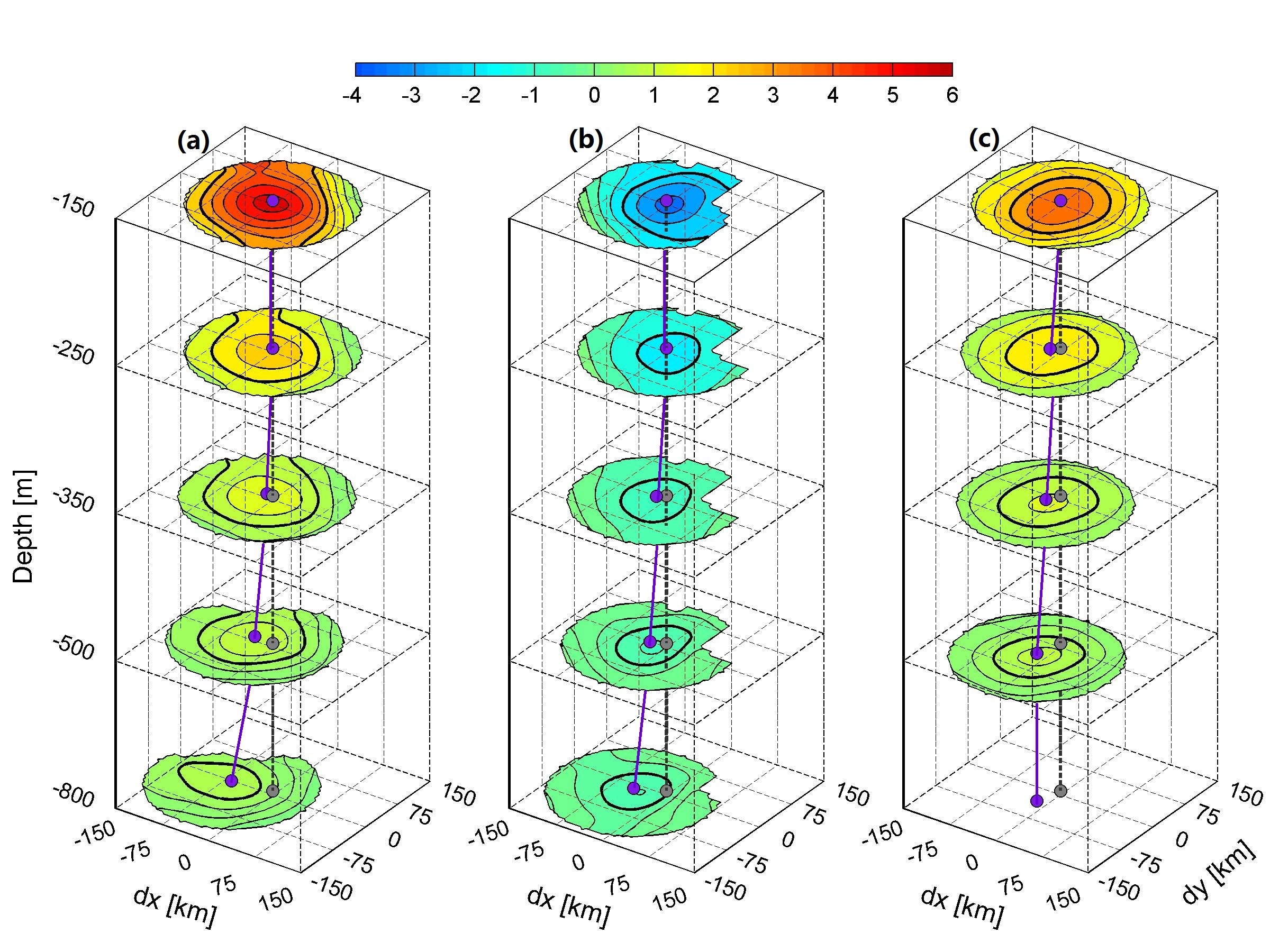


**Supplementary Figure S7 | 3D temperature structure of oceanic eddies.** 3D structures of temperature anomaly (shadings, unit in ℃) for **(a)** AE0, **(b)** CE and **(c)** AE1. For a better visual effect, temperature anomaly amplitudes at 500 and 800 m have been amplified by a factor of 1.5 and 2, respectively. The contour intervals (black lines) at 150, 250, 350, 500 and 800 m are 0.6, 0.6, 0.3, 0.15, 0.15 ℃, respectively. The black thick lines at layers from top to bottom indicate the contours of 3, 1.8, 0.9, 0.6 and 0.6 ℃, respectively for (a) and (c), and 1.8, 1.2, 0.6, 0.45 and 0.45 ℃, respectively for (b). Eddy centers at each layer, which are defined at the point of maximum temperature anomaly, are indicated by the purple dots. Purple line denotes the axis of eddy, defined as the line connecting the eddy centers at each layer. Gray dots and dashed line indicate the z-axis of the coordinate. Figures are plotted using MATLAB R2013a (http://www.mathworks.com/).


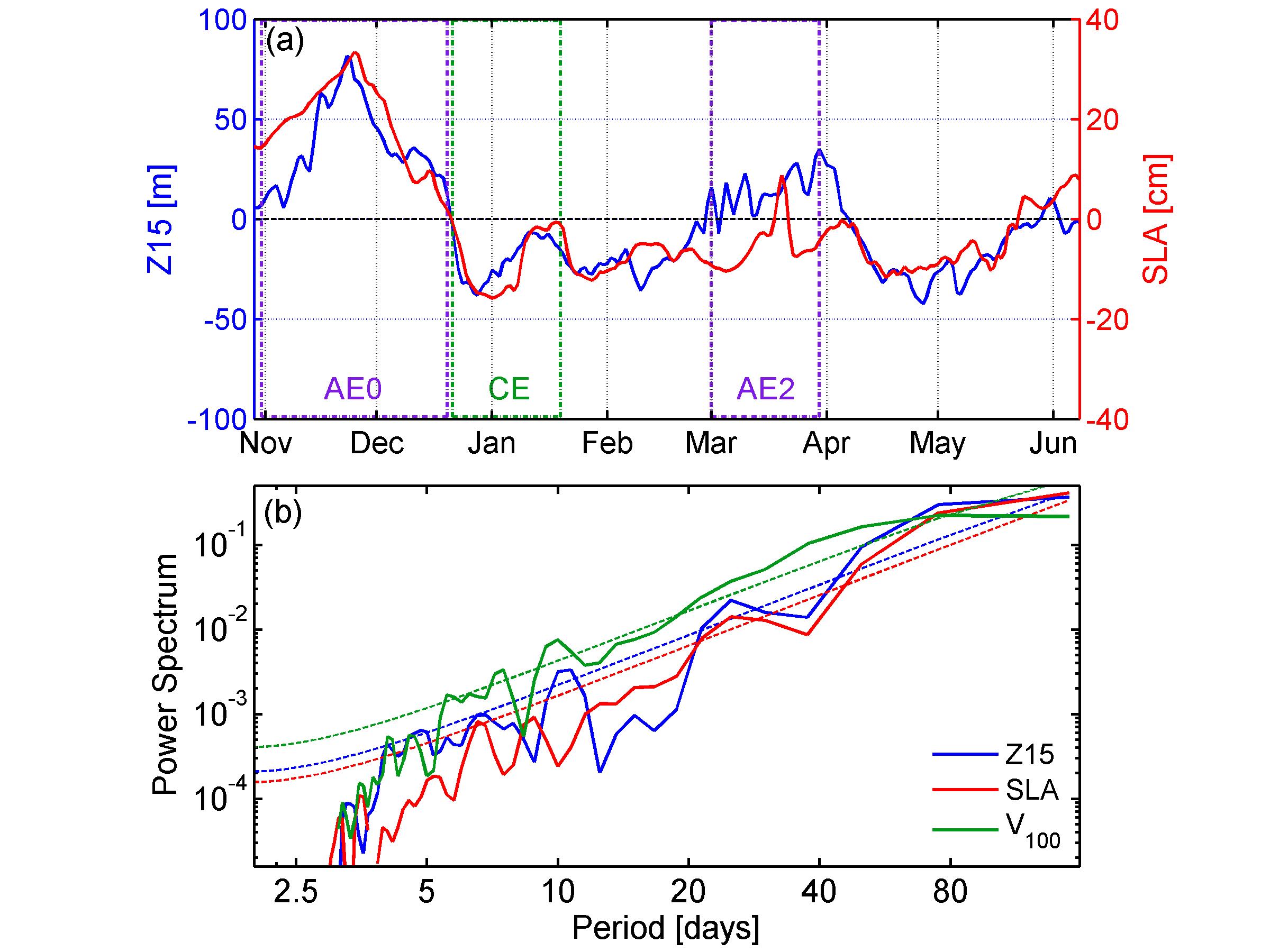


**Supplementary Figure S8 | Time series and power spectrum. (a)** Time series of the depth of 15℃ (Z15, blue line) and the SLA (red line) at the mooring site M9. The first purple box, the green box and the second purple box indicate the periods of AE0, CE and another anticyclonic eddy AE2, respectively. **(b)** Power spectrum of the Z15 (blue solid line) and SLA (red solid line) shown in **(a)**. Also shown is the power spectrum of the meridional velocity at depth of 100 m (V_100_). The dashed lines denote the 95% confidence level of the corresponding variability based on AR1 red noise spectrum. Figures are plotted using MATLAB R2013a (http://www.mathworks.com/).


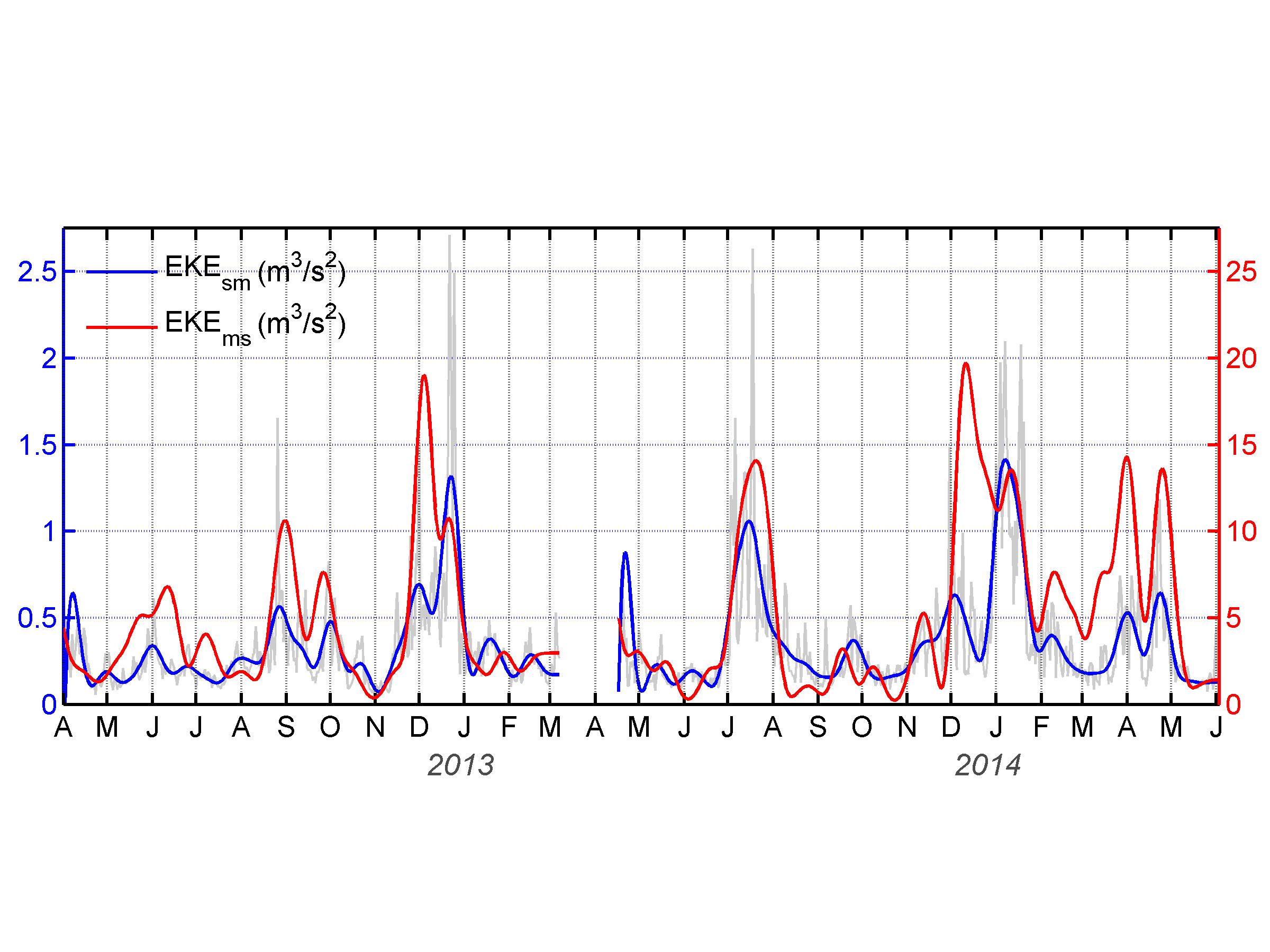


**Supplementary Figure** **S9 | Time series of the kinetic energy at mooring site M1** **from April 2012 to June 2014.** The gray line is the depth-integrated EKE_sm_ and the blue line is the corresponding 20-day low-pass filtered result. The red line denotes the 20-day low-pass filtered EKE_ms_. The correlation coefficient is 0.66 (above the 95% significance level). Figure is plotted using MATLAB R2013a (http://www.mathworks.com/).


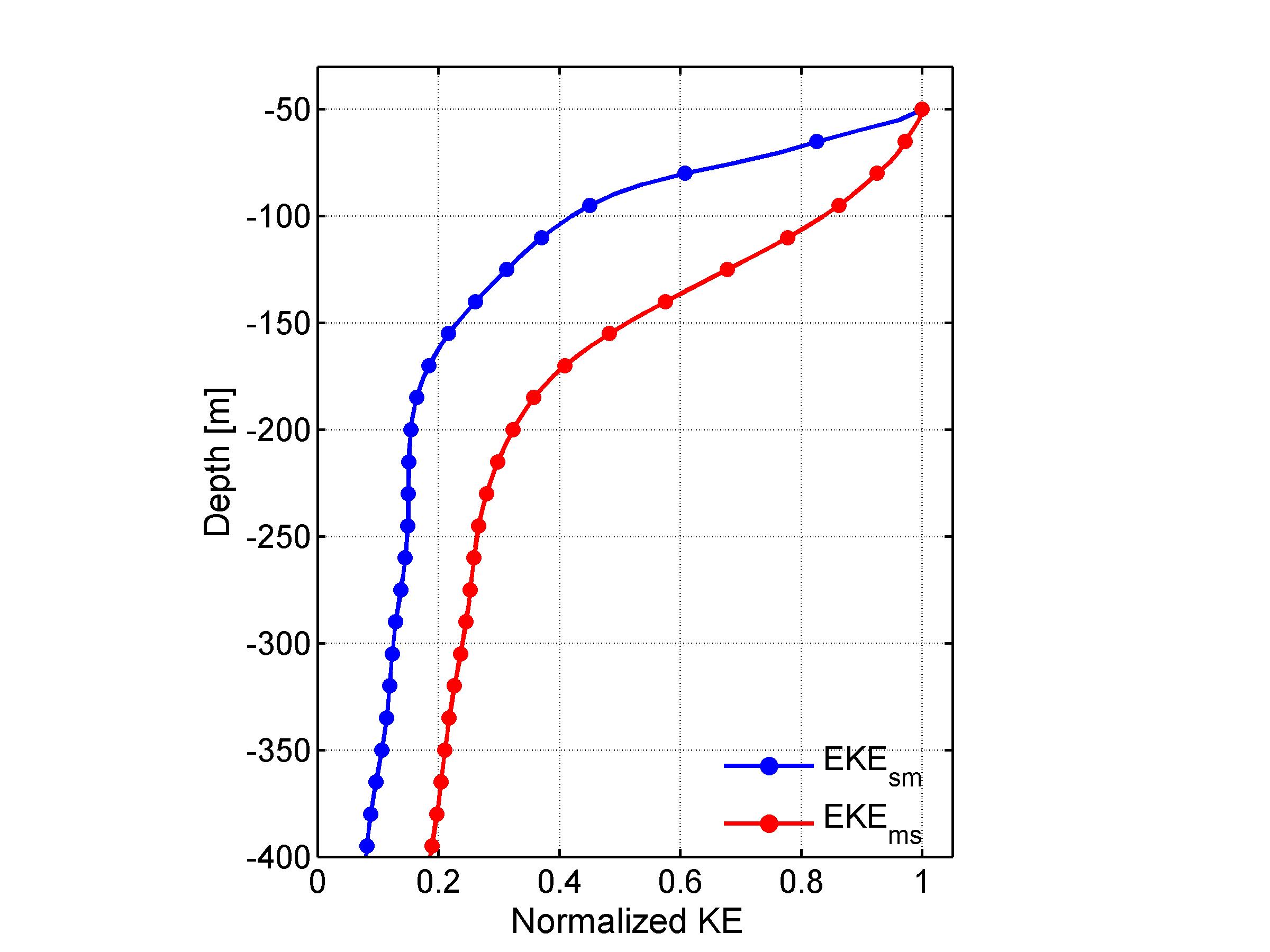


**Supplementary Figure S10 | Mean vertical structure of the kinetic energy at mooring site M1.** Blue and red dotted lines denote the EKE_sm_ and EKE_ms_, respectively. The result is normalized by dividing its maximum value. The mean profiles are calculated based on data from April 2012 to June 2014. Figure is plotted using MATLAB R2013a (http://www.mathworks.com/).


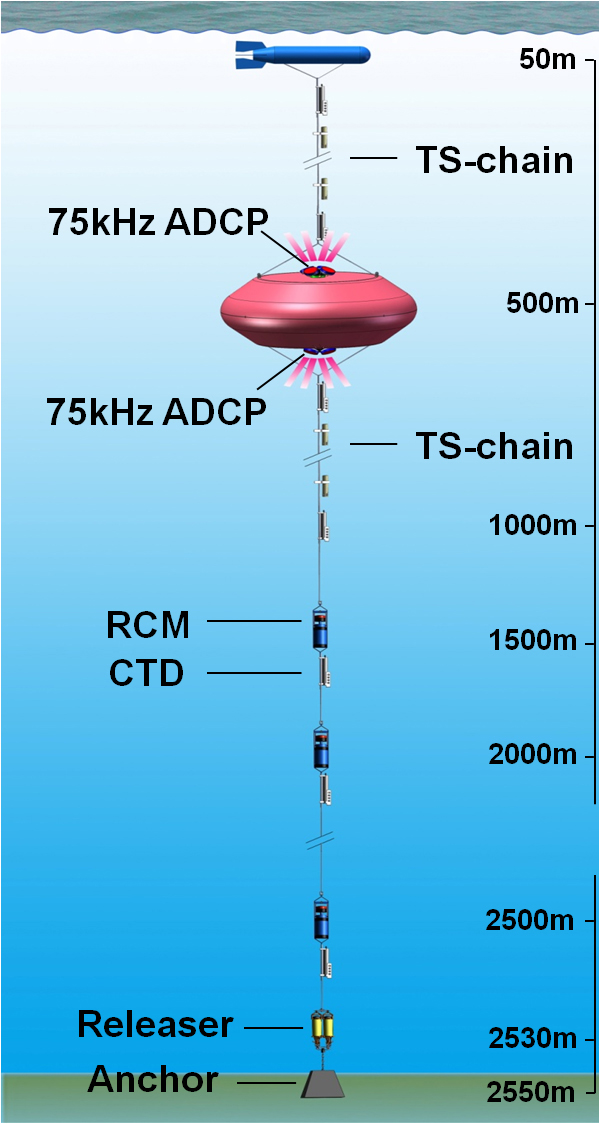


**Supplementary Figure S11 | Schematic diagram of the configuration of a subsurface mooring.** The names of the instruments and their vertical locations are marked**.** The water depth is 2550 m.

**Supplementary Table S1 | Detailed configuration of the moorings deployed during the S-MEE ^a^**.

| Mooring | Observation Period | Longitude, Latitude | Water Depth (m) | Instrument | Instrument Depth (m) | Range Depth (m) | Bin Size (m) | Sample Interval (min) |
| --- | --- | --- | --- | --- | --- | --- | --- | --- |
| M1 | Aug/18/2013–Jun/03/2014 | 117.87°E, 21.11°N | 953 | Temperature chains | ** | 75-435 | 20 | 3 |
|  |  |  |  | 75 kHz ADCP (up)  75 kHz ADCP (dw) | 435  435 | 45-413  451-883 | 16  16 | 3 |
|  |  |  |  | CTDs | 75, 175, 375 | ** | ** | 3 |
| M2 | Oct/21/2013– Jun/03/2014 | 118.16°E, 21.01°N | 2021 | Temperature chains | ** | 65-465  465-665  665-965 | 20  50  100 | 2 |
|  |  |  |  | 75 kHz ADCP (up)  75 kHz ADCP (dw)  75 kHz ADCP (dw)  300 kHz ADCP (dw) | 480  480  950  1920 | 48-464  496-944  965-1381  1935-2015 | 16  16  16  4 | 3 |
|  |  |  |  | RCMs | 1696 | ** | ** | 30 |
|  |  |  |  | CTDs | 65, 265, 465, 965, 1701, 1915 | ** | ** | 2 |
| M3 | Oct/27/2013– Jun/02/2014 | 118.58°E, 20.91°N | 2520 | Temperature chains | ** | 75-95  95-475  475-675  675-975 | 10  20  50  100 | 2 |
|  |  |  |  | 75 kHz ADCP (up)  75 kHz ADCP (dw) | 475  475 | 45-461  496-960 | 16  16 | 3 |
|  |  |  |  | RCMs | 1514, 2021, 2430 | ** | ** | 30 |
|  |  |  |  | CTDs | 75, 115, 175, 275, 475, 975, 1519, 2026, 2435 | ** | ** | 2 |
| M4 | Oct/28/2013– Jun/01/2014 | 118.92°E, 20.25°N | 2918 | Temperature chains | ** | 65-85  85-465  465-665  665-965 | 10  20  50  100 | 2 |
|  |  |  |  | 75 kHz ADCP (up)  75 kHz ADCP (dw) | 465  465 | 40-456  485-965 | 16  16 | 3 |
|  |  |  |  | RCMs | 1501, 2014, 2420, 2828 | ** | ** | 30 |
|  |  |  |  | CTDs | 65, 105, 165, 265, 465, 965, 1506, 2019, 2425, 2833 | ** | ** | 2 |
| M5 | Oct/28/2013– Jun/23/2014 | 118.97°E, 20.57°N | 2770 | Temperature chains | ** | 60-130  130-510  510-710  710-1010 | 10  20  50  100 | 2 |
|  |  |  |  | 75 kHz ADCP (dw) | 110 | 121-761 | 16 | 60 |
|  |  |  |  | RCMs | 1549, 2574 | ** | ** | 30 |
|  |  |  |  | CTDs | 60, 130, 230, 510, 1010, 1554, 2578 | ** | ** | 2 |
| M6 | Apr/24/2013– Jun/03/2014 | 119.05°E, 20.83°N | 2759 | 75 kHz ADCP (up)  75 kHz ADCP (dw) | 505  505 | 42-490  531-995 | 16 | 3 |
|  |  |  |  | RCMs | 1547, 2059, 2676 | ** | ** | 60 |
|  |  |  |  | CTDs | 500, 1552, 2064, 2681 | ** | ** | 3 |
| M7 | Oct/28/2013– Jun/07/2014 | 119.11°E, 21.25°N | 2787 | Temperature chains | ** | 60-80  80-460  460-660  660-960 | 10  20  50  100 | 2 |
|  |  |  |  | 75 kHz ADCP (up) | 475 | 45-461 | 16 | 3 |
|  |  |  |  | RCMs | 991, 1494, 2001, 2510, 2716 | ** | ** | 30 |
|  |  |  |  | CTDs | 60, 100, 160, 460, 996, 1499, 2006, 2515, 2721 | ** | ** | 2 |
| M8 | Oct/27/2013– Jun/06/2014 | 119.23°E, 21.70°N | 2561 | Temperature chains | ** | 110-130  130-510  510-710  710-1010 | 10  20  50  100 | 3 |
|  |  |  |  | 75 kHz ADCP (up)  75 kHz ADCP (dw) | 510  510 | 53-485  530-994 | 16 | 2 |
|  |  |  |  | RCMs | 1541, 2050, 2462 | ** | ** | 30 |
|  |  |  |  | CTDs | 110, 150, 210, 510, 1010, 1546, 2055, 2467 | ** | ** | 3 |
| M9 | Oct/29/2013– Jun/08/2014 | 119.66°E, 20.69°N | 3092 | Temperature chains | ** | 60-80  80-460  460-660  660-960 | 10  20  50  100 | 2 |
|  |  |  |  | 75 kHz ADCP (up)  75 kHz ADCP (dw) | 460  460 | 43-443  473-921 | 16 | 3 |
|  |  |  |  | RCMs | 1482, 1996, 2494, 3004 | ** | ** | 30 |
|  |  |  |  | CTDs | 60, 100, 160, 460, 960, 1487, 2001, 2499, 3009 | ** | ** | 2 |
| M10 | Oct/29/2013– Jun/09/2014 | 120.22°E, 20.57°N | 3847 | Temperature chains | ** | 160-180  180-560  560-760  760-1060 | 10  20  50  100 | 2 |
|  |  |  |  | 75 kHz ADCP (up)  75 kHz ADCP (dw) | 560  560 | 58-538  578-994 | 16 | 3 |
|  |  |  |  | RCMs | 1565, 2175, 2782, 3289, 3803 | ** | ** | 30 |
|  |  |  |  | CTDs | 160, 200, 260, 560, 1060, 1570, 2180, 2787, 3294, 3808 | ** | ** | 2 |
| M11 | Jan/14/2014– Jun/19/2014 | 115.29°E, 20.22°N | 480 | Temperature chains | ** | 55-255  255-455 | 20  50 | 1/3 |
|  |  |  |  | 150 kHz ADCP (up) | 265 | 42-250 | 8 | 3 |
|  |  |  |  | RCMs | 396 | ** | ** | 3 |
|  |  |  |  | CTDs | 55, 405 | ** | ** | 1/3 |
| M12 | Jan/13/2014– Jun/19/2014 | 115.31°E, 20.07°N | 898 | Temperature chains | ** | 60-360  360-460  460-660 | 20  50  100 | 1/6 |
|  |  |  |  | 75 kHz ADCP (up) | 460 | 56-440 | 16 | 3 |
|  |  |  |  | RCMs | 565, 765 | ** | ** | 3 |
|  |  |  |  | CTDs | 60, 560 | ** | ** | 1/6 |
| M13 | Jan/13/2014– Jun/19/2014 | 115.37°E, 19.85°N | 1565 | Temperature chains | ** | 35-335  335-435  435-1035 | 20  50  100 | 1/6 |
|  |  |  |  | 75 kHz ADCP (up)  300 kHz ADCP (dw) | 455  1465 | 40-440  1478-1554 | 16  4 | 3  15 |
|  |  |  |  | RCMs | 557, 900, 1100, 1300 | ** | ** | 3 |
|  |  |  |  | CTDs | 35, 335, 905, 1468 | ** | ** | 1/6 |
| M14 | Jan/13/2014– Jun/19/2014 | 115.46°E, 19.54°N | 2298 | Temperature chains | ** | 15-315  315-415  415-515  515-1515 | 20  50  100  200 | 1/6 |
|  |  |  |  | 75 kHz ADCP (up) | 435 | 50-418 | 16 | 3 |
|  |  |  |  | RCMs | 571, 1020, 1573, 2088 | ** | ** | 3 |
|  |  |  |  | CTDs | 15, 315, 1025, 2093 | ** | ** | 1/6 |
| M15 | Jan/13/2014– Jun/20/2014 | 115.53°E, 19.19°N | 2606 | Temperature chains | ** | 25-325  325-425  425-525  525-1525 | 20  50  100  200 | 1/6 |
|  |  |  |  | 75 kHz ADCP (up) | 435 | 45-413 | 16 | 3 |
|  |  |  |  | RCMs | 562, 1045, 1576, 2093 | ** | ** | 3 |
|  |  |  |  | CTDs | 25, 325, 1050, 2098 | ** | ** | 1/6 |
| M16 | Jan/13/2014– Jun/21/2014 | 115.65°E, 18.79°N | 3313 | Temperature chains | ** | 25-225  225-425  425-625 | 20  50  100 | 1/3 |
|  |  |  |  | RCMs | 60, 558, 1049, 1558, 2078, 2577, 3090 | ** | ** | 3 |
|  |  |  |  | CTDs | 25, 625, 1563, 3095 | ** | ** | 1/3 |
| M17 | Jan/12/2014– Jun/21/2014 | 115.77°E, 18.32°N | 3783 | Temperature chains | ** | 35-235  235-435  435-635 | 20  50  100 | 1/3 |
|  |  |  |  | RCMs | 70, 604, 1070, 1604, 2104, 2604, 3204 | ** | ** | 3 |
|  |  |  |  | CTDs | 35, 635, 1075, 3209 | ** | ** | 1/3 |

^a^ The ADCPs are manufactured by the TRDI Company. The RCMs are Seaguard current meters manufactured by Aanderaa. The CTDs are of Seabird 37SM. ‘Up’ and ‘dw’ indicate ADCPs looking upward and downward, respectively. The temperature chain consists of 2–6 CTDs and dozens of thermometers (Seabird SBE56) depending on different mooring site. Vertical positions of the CTDs located in the temperature chains are included in the 'Instrument Depths'.
